# Effects of a psychoeducational group intervention for adults diagnosed with ADHD: a pilot randomized controlled study

**DOI:** 10.1186/s12888-025-07452-5

**Published:** 2025-10-14

**Authors:** Åshild Holsbrekken, Tatiana Skliarova, Arthur Mandahl, Sverre Georg Sæther, Anne Grethe Sjøbakk Lund, Jasna Surkovic, Erik Søndenaa, Jonas Vaag, Terje Torgersen, Mariela L. Lara-Cabrera

**Affiliations:** 1 Division of Psychiatry, Nidaros Community Mental Health Center, St. Olav’s University Hospital, Trondheim, Norway; 2 Department of Mental Health, Faculty of Medicine and Health Sciences, Norwegian University of Science and Technology (NTNU), Trondheim, Norway; 3 Vårres Regional User-Led Center Mid-Norway, Trondheim, Norway; 4 Blue Cross Lade Addiction Treatment Centre, Trondheim, Norway; 5 Faculty of Nursing and Health Science, Nord University, Namsos, Norway; 6 Department of Psychiatry, Levanger Hospital, Nord-Trøndelag Hospital Trust, Levanger, Norway; 7 Department of Mental Healthcare, St. Olav’s Hospital, Nidelv Community Mental Health Centre, Trondheim University Hospital, Trondheim, Norway; 8 Center for Research and Education in Forensic Psychiatry, St. Olav’s Hospital, Trondheim, Norway; 9 Department of Psychology, University of Inland Norway, Lillehammer, Norway

**Keywords:** Attention-deficit/hyperactivity disorder (ADHD), Group intervention, Patient-centred care, Patient education, Peer co-facilitated education, Psychoeducational intervention, Quality of life, Randomized controlled trial (RCT), User involvement

## Abstract

**Introduction:**

Psychoeducational group programs have shown promising results for people with attention-deficit/hyperactive disorders (ADHD). This pilot study aimed to investigate the impact of a new co-produced psychoeducational group intervention on quality of life (QoL), ADHD-related symptoms, and patient satisfaction.

**Method:**

This pragmatic pilot randomized controlled trial (RCT) had two parallel arms. Outpatients were recruited in two community mental health centers (CMHCs) in the Central Norway Regional Health Authority. Patients eligible for inclusion were diagnosed with ADHD. The psychoeducation group (PG) received a 10-session psychoeducational group program as a supplement to treatment as usual (TAU), while the control group (CG) received TAU only. The program was developed in collaboration between user representatives and health professionals. While the primary aim of this pilot study was to assess the preliminary impact of the intervention, we conducted exploratory between-group comparisons to identify potential effects on patient-centered outcomes. The primary outcome was QoL measured with the Adult ADHD Quality of Life Scale (AAQoL). Secondary outcomes were ADHD symptom severity (Adult Self-Report Scale, 6-Item) and patient satisfaction (Satisfaction with Information on ADHD and Treatment Scale). We collected data at baseline, pre-, and post-intervention (T0, T1, and T2 respectively).

**Results:**

Of 49 patients, 27 were allocated to the PG and 22 to the CG. Between-group comparisons using linear mixed models indicated a statistically significant improvement in QoL (mean difference = 6.90; *p* = 0.04; 95% CI [0.20–13.60]; Cohen’s *d* = 0.49). Outpatients in the PG reported significantly higher satisfaction with information as compared to the CG (mean difference = 2.14; *p* = 0.02; 95% CI [0.33–3.95]; Cohen’s *d* = 0.78). However, there were no significant differences in ADHD symptoms between the groups.

**Conclusion:**

This psychoeducational group intervention significantly improved QoL and patient satisfaction but not symptom burden in adult patients with ADHD. Future studies should explore the long-term effects of psychoeducational group programs and examine ways to optimize treatment outcomes.

**Trial registration:**

ClinicalTrials.gov NCT03337425, 06/11/2017.

**Supplementary Information:**

The online version contains supplementary material available at 10.1186/s12888-025-07452-5.

## Background

ADHD is a neurodevelopmental disorder characterized by persistent challenges with attention, hyperactivity, and impulsivity from childhood [1]. Although the diagnosis is mainly made in children, the diagnostic criteria have changed over time so that the diagnosis is now also made in adults [2]. An estimated 2.5%–5% of adults have a persistent ADHD diagnosis from childhood, and about 7% exhibit symptoms consistent with an ADHD diagnosis in adulthood [3–6].

Being diagnosed with ADHD in adulthood can be challenging [7]. The diagnosis encompasses more than just the core symptoms of ADHD; it can also result in challenges with executive functions and impaired adaptive functioning, which can impact relationships [8], difficulties with education and working life [9], and overall poorer functioning in daily life [10]. This can lead to a poorer quality of life (QoL) for people who have been diagnosed with ADHD [11]. In addition, it is known that many who have the diagnosis also have additional problems, such as comorbid mental and substance use disorders [12], behavioral addictions [13], stress, lack of social support, physical illness, financial difficulties, unemployment, and early death [14]. Thus, there is a need for a broad perspective on treatment interventions for ADHD. Further, pharmacological interventions are the first-line treatment in adult ADHD [15], however, not everyone benefits from medication [16–18]. In fact, adult ADHD patients have a low adherence and high dropout rate from outpatient treatment [19]. This highlights the importance of developing tailored treatment methods for this patient group. Given the complexity and diverse challenges they face, it is essential to create a more differentiated service.

The World Health Organization (WHO) describes quality of life as “individuals’ perception of their position in life, in the context of culture and value systems in which they live, and in relation to their goals, expectations, standards, and concerns” [20]. In recent research, greater emphasis has been placed on measuring changes in QoL [21]. It may be argued that QoL is a more relevant and important outcome than ADHD symptoms per se [22]. Previous studies have indicated that adults diagnosed with ADHD have a lower QoL compared to those without ADHD [23–25]. This is evident in various aspects of life, including physical health, mental health, relationships, and overall life outcomes [26]. Studies have also shown that individuals with ADHD have a lower level of education, lower employment, lower wage levels, and greater financial difficulties compared to people without ADHD [27–30]. Additionally, older people with the diagnosis are more likely to develop depression and loneliness [31]. However, few treatment-effect studies use QoL as the primary outcome measure.

ADHD patients need and want to learn about various aspects of the diagnosis [14, 32]. They seek comprehensive information covering the diagnosis itself, its impact on daily life, and strategies for managing the associated challenges [33–35]. Providing information to patients who struggle with health literacy is essential for their engagement in treatment [36] and their ability to make informed decisions regarding treatment options [34]. Despite this, efficacy studies of patient information and user participation interventions for adults with ADHD are sparse [37].

Providing targeted information about specific diagnoses has become crucial to treatment in most healthcare settings. In psychiatry, systematic reviews have demonstrated that psychoeducation has been beneficial for individuals with long-term illnesses such as schizophrenia [38] and bipolar disorder [39]. However, little is known about the effect of psychoeducation groups on adults with ADHD. Skliarova et al. [40] did a scoping review on the subject that concluded that psychoeducation in groups as an intervention is beneficial for adults with an ADHD diagnosis. However, research examining the quality and satisfaction of patients with the information received is necessary to improve the provision of such services.

Recent studies suggest that involving health service users and former patients in both the development and evaluation processes is beneficial [41, 42]. The participation of those who use the services is important to ensure that the intervention design reflects the needs and preferences of participants, thereby increasing the likelihood that the intervention is valid from an end-user perspective [43]. Research indicates that user involvement and co-creation positively impact both research processes and individual experiences [44], enhancing health-related outcomes at the individual level and benefiting public health overall [42, 45, 46]. However, few studies have considered the user perspective when developing new interventions for adults with ADHD [47], and only one study has included former patients in intervention delivery [48]. Research involving users in the development and delivery of psychoeducational programs is thus at an early stage and requires further study.

Psychoeducational group programs for adults diagnosed with ADHD have shown promising results in several RCTs [40]. Vidal et al. [49] compared psychoeducation groups and CBT groups for adults with ADHD and found improvement in ADHD symptoms in both groups. Hirvikoski et al. [7] determined that, compared to patients receiving traditional treatment, patients receiving the psychoeducational group intervention experienced many benefits. Patients in the psychoeducational program reported a decrease in psychological stress and ADHD symptoms, in addition to reporting an increase in knowledge about ADHD and QoL. De Braek et al. [50] studied the effects of psychoeducation alone compared to combined psychoeducation and goal management training, and concluded that both groups had significant improvement in function, with the intervention groups showing the greatest change. Additionally, Hoxhaj et al. [51] compared the effects of psychoeducation groups to those of mindfulness groups, reporting that both interventions resulted in significant improvements of QoL and decreased ADHD-related symptoms. Similarly, Bachman et al. [52] studied the effects of psychoeducation and mindfulness groups, reporting that the interventions resulted in improvements in working memory and ADHD symptoms.

In more recent studies, Selaskowski et al. [53] examined the effects of psychoeducation through an app versus written material. Both groups significantly improved ADHD symptoms, but the patients receiving psychoeducation through the app had the greatest improvement. Skliarova et al. [48] investigated the feasibility, acceptability, and preliminary evaluation of a psychoeducation group intervention in an outpatient setting. Their pilot RCT study showed feasibility, reporting low dropout rates, high attendance, and an improvement in satisfaction with information and QoL. Finally, Skliarova et al. [54] found in their pilot RCT study that a two-session peer-cofacilitated psychoeducational program led to significant improvement in satisfaction with the information.

Despite these encouraging findings, recent scoping and systematic reviews [40, 47, 54] have highlighted several persistent gaps in evidence-based psychoeducational group interventions for adults with ADHD. First, relatively few studies have focused exclusively on psychoeducation as a standalone intervention, which has hindered efforts to synthesize findings through meta-analyses. Second, although there is a growing consensus on the value of including individuals with lived experience in research, particularly in the design and delivery of interventions, this participatory approach remains rare [47]. To date, only a few published studies have involved adults with ADHD in the development of psychoeducational content. In addition, few trials in this area have assessed patient-centered outcomes such as QoL [7, 48, 49, 51]. These outcomes are closely linked to patients’ subjective experiences of outpatient treatment but are not consistently reported, limiting our understanding of the broader impact of psychoeducational programs.

To help address these gaps, we conducted a pilot RCT of a novel psychoeducational program for adults with ADHD. The intervention was co-developed and co-delivered by service users and clinicians in equal partnership. This participatory model aims to improve the relevance, acceptability, and potential impact of the program by carrying out exploratory between-group comparisons to inform the design of future trials. The primary outcome is to assess changes in QoL. We hypothesized that patients receiving the intervention would show a significant improvement in QoL compared to controls. The secondary outcome is exploring differences in ADHD symptoms and satisfaction with information between groups.

## Method

### Design and setting

This pragmatic, two-center, parallel, two-armed pilot randomized controlled trial (RCT) investigated the pre-post effects of a psychoeducation group intervention compared to a control group receiving TAU. The study protocol has been published previously [55]. The study’s design and the reporting of results were conducted in accordance with the Consolidated Standards of Reporting Trials (CONSORT) guidelines [56]. To ensure that the study is reported accurately, the CONSORT checklist for pilot studies has been followed and is available in the supporting material [57].

The psychoeducational intervention was given in two community mental health centers in the Central Norway Regional Health Authority. This trial was approved by the Ethics Committee and subsequently pre-registered with ClinicalTrials.gov (NCT03337425). This article is part of a larger project focused on psychoeducation in groups for patients diagnosed with ADHD in adulthood. Previously, a feasibility, acceptability, and preliminary evaluation study [48] was conducted using data from one of the centers (*n* = 30).

### Recruitment, procedures, and eligibility criteria

Recruitment for the pilot RCT followed a structured procedure designed to ensure that eligible participants met the necessary criteria and were fully informed before enrolling. Recruitment took place between November 2017 and March 2018. Patients diagnosed with ADHD, who received specialized outpatient treatment were eligible for participation. Recruitment materials, including one-page flyers describing the study, were distributed by therapists to their patients during their regular clinical sessions. Outpatients were invited to attend an in-person information meeting. This in-person meeting at the clinic provided an opportunity for the research team to explain the study in more detail, answer any questions the patients had, and assess their eligibility based on the inclusion or exclusion criteria.

Inclusion criteria for participants were defined based on a specific target population. Participants had to be between 18 and 67 years old, fluent in a Scandinavian language (i.e., Norwegian, Swedish, or Danish), and diagnosed with ADHD. Furthermore, candidates for participation had to indicate that they were willing to participate in the study as well as commit to attending group therapy sessions. Patients were only invited to participate if they were free of psychotic disorders or severe learning disabilities and could provide informed consent. Not being able to consent in an informed way could be due to severe cognitive or memory disorders or declarations of legal incompetence. These measures were implemented to ensure that only those individuals who could fully understand the implications of their participation were included. Patients already involved in other research studies or group therapy sessions for ADHD were also excluded. This was done to filter out potentially overlapping therapy or interventions, which may have affected the study’s outcomes. Patients eligible for inclusion received both written and oral descriptions of the study, including study objectives, procedures, their right to withdraw from the study without any negative consequences for their treatment, and potential risks and benefits from study participation.

### Sample size and randomization

This study was conducted as a pilot study, and between-group comparisons were conducted. A formal a priori sample size calculation for the primary outcome, QoL, was not carried out. Instead, the sample size was based on recommendations for pilot studies, which suggest including at least 12 participants per group [58]. We aimed to invite a total of 60 participants. Eligible patients who provided written informed consent for study participation were randomized. Participants were then randomized to either the psychoeducation group (PG) or the control group (CG) with a 1:1 allocation ratio. A block randomization procedure was used to allocate the two groups via independent computer-assisted software [55].

### Treatment arms: intervention versus control group

The intervention consisted of 10 sessions conducted over the course of 10 weeks. Each session focused on a specific topic, inviting an expert to deliver a lecture lasting approximately 20 min. After the lecture, a discussion of approximately 45 min followed, which was led by the course leader. The experts giving the lectures were from clinical settings, including professionals such as social workers, psychologists, medical doctors, and psychiatrists, as well as representatives from user organizations. A script was developed on each topic and used by the lecturers, ensuring that the same information was given to all participants. The course covered the following topics: myths and facts about ADHD, the definition of ADHD, ADHD and comorbidities, medication use, economic implications, self-help groups, daily life coping strategies, and work and welfare. The CG TAU, which for most patients included pharmacological treatment and/or psychotherapy as needed. If needed, they also received follow-up in relation to finances, housing, work, or education, or help with interacting with family and networks. The CG was offered the psychoeducational group program after the study period had ended.

### Demographic data and measures

Demographic information was completed at baseline (T0), while the outcome measurements were collected at three different points in time, assessing pre-intervention stability, as well as capturing change over time. All participants completed the baseline measurement prior to randomization. The second measurement (T1) was administered immediately before the first psychoeducation session (for PG) or at an equivalent time point for CG. This design allowed us to assess pre-intervention stability. The final measurement (T2) was collected 10 weeks after T1 for both groups. During this period, PG received the intervention, while the CG continued with TAU only. See Table 1 for an overview of data collection, questionnaires, measurement points, and their internal consistency measures.

**Table 1:** Data collection and measurement points

Data gathered	Timepoints			Cronbach’s alpha
	T0	T1	T2	
Sociodemographic	X			
AAQoL	X	X	X	0.923
ASRS-6	X	X	X	0.620
SIATS	X	X	X	0.827

*T0* Time 0, baseline, *T1* Time 1, pre-intervention, *T2* Time 2, post-intervention, *AAQoL* Adult ADHD Quality of Life Scale, *ASRS-6* Adult ADHD Self-Reported Scale, 6 Items, *SIATS* Satisfaction with Information on ADHD and Treatment Scale

### Sociodemographic data

Sociodemographic information about the participants was collected via self-report at T0. The data gathered included age, sex, relationships, education level, employment, and housing situation.

### Primary outcome

Despite not powering the RCT pilot study, the primary measure regarding QoL was identified a priori. This primary outcome measure was the Adult ADHD Quality of Life Scale (AAQoL). This is a self-report questionnaire that measures the patient’s perception of the emotional and functional impact of ADHD on their quality of life. It provides a score based on 29 questions divided into four subgroups: life productivity, psychological health, relationships, and overall life outcomes [59, 60]. The 29 items are rated on a 5-point Likert scale. In this study, the validated Norwegian version was used [11]. See Table 1 for data collection and measurement points.

### Secondary outcomes

The first secondary outcome was ADHD symptoms, assessed using the six-item Adult ADHD Self-Report Scale-6 (ASRS-6). The ASRS is a self-rated scale developed based on the symptoms that form the basis for the diagnosis described in the DSM-IV. While the original scale consists of 18 questions [61], we selected the shorter ASRS-6 to reduce participant burden, particularly given the attention difficulties in this population [62]. Several studies have demonstrated that the ASRS-6 has good psychometric properties in research context [63–65]. The six questions on the scale are rated from 0 to 4, where 0 equals “never,” 1 “rarely,” 2 “sometimes,” 3 “Often,” and 4 “very often.” See Table 1 for data collection.

The other secondary outcome was patients’ satisfaction with information assessed using the Satisfaction with Information on ADHD and Treatment Scale (SIATS). SIATS is a three-item measure focusing on participants’ perceptions of the quality and clarity of the information they received during psychoeducation intervention [34]. Specifically, the items measured participants’ satisfaction with information on three key aspects of their condition and treatment: information about ADHD, information about treatment options, and information about pharmacological treatment. Each of these items was rated on a 5-point Likert scale, where patients rated their satisfaction from 1 (“not satisfied”) to 4 (“very satisfied”); a score of 5 indicated “I don’t know.” The total score ranges from 3 to 12, representing the highest level of satisfaction across all three items.

### Statistical methods and masking

Data analyses were performed on intention to treat samples using SPSS version 28.0.1.0 [66]. Descriptive statistics were used to summarize demographic baseline data and questionnaires, reporting the mean (M), standard deviation (SD), and proportions. Baseline differences between the PG and the CG were examined using independent sample *t*-test. The chi-squared test of associations or Fisher’s exact test were used to compare categorical variables. This ensured that differences in group proportions were tested thoroughly.

The primary outcome measure, AAQoL, and secondary outcomes, ASRS-6 and SIATS, were analyzed using data collected from T0, T1, and T2. Although both T0 and T1 occurred before the intervention, participants were treated analytically as a single cohort during this phase to establish a stable pre-intervention baseline. This approach enabled us to isolate the potential impact of the psychoeducation program by focusing on changes between T1 (pre-intervention) and T2 (post-intervention).

While the primary aim of this pilot study was to assess the preliminary impact of the intervention, we conducted exploratory between-group comparisons using linear mixed models to identify potential effects on patient-centered outcomes. Linear mixed models were employed to account for repeated measures and accommodate missing-at-random data.

We used linear mixed model analysis to find between-group differences in outcomes at different time points. In the model, the combination of time point and treatment group (with levels “Pre-randomization,” “Pre-intervention,” “Psychoeducation Group [PG],” and “Control Group [CG]”) was treated as a fixed effect, while participant ID was treated as a random effect to account for individual variability. Cohen’s *d* was applied to estimate between-group effect sizes, interpreted as small (0.2), medium (0.5), or large (≥ 0.8) [67, 68].

An intention-to-treat (ITT) analysis was performed, meaning that all randomized outpatients were included in the analysis [69]. As such, ITT ensures that all randomized participants were included in the analyses according to their assigned groups, regardless of intervention adherence or missing data, and there is no need for multiple imputations [70]. Statistical significance was set at *p* < 0.05.

Blinding the participants was not possible, but the person conducting the statistical analyses was blinded to group allocation while conducting the SPSS analyses. The masking was done by assigning dummy codes to the two groups to minimize any biases that could arise from knowing which participants were in the CG or PG.

## Results

**Fig. 1:**
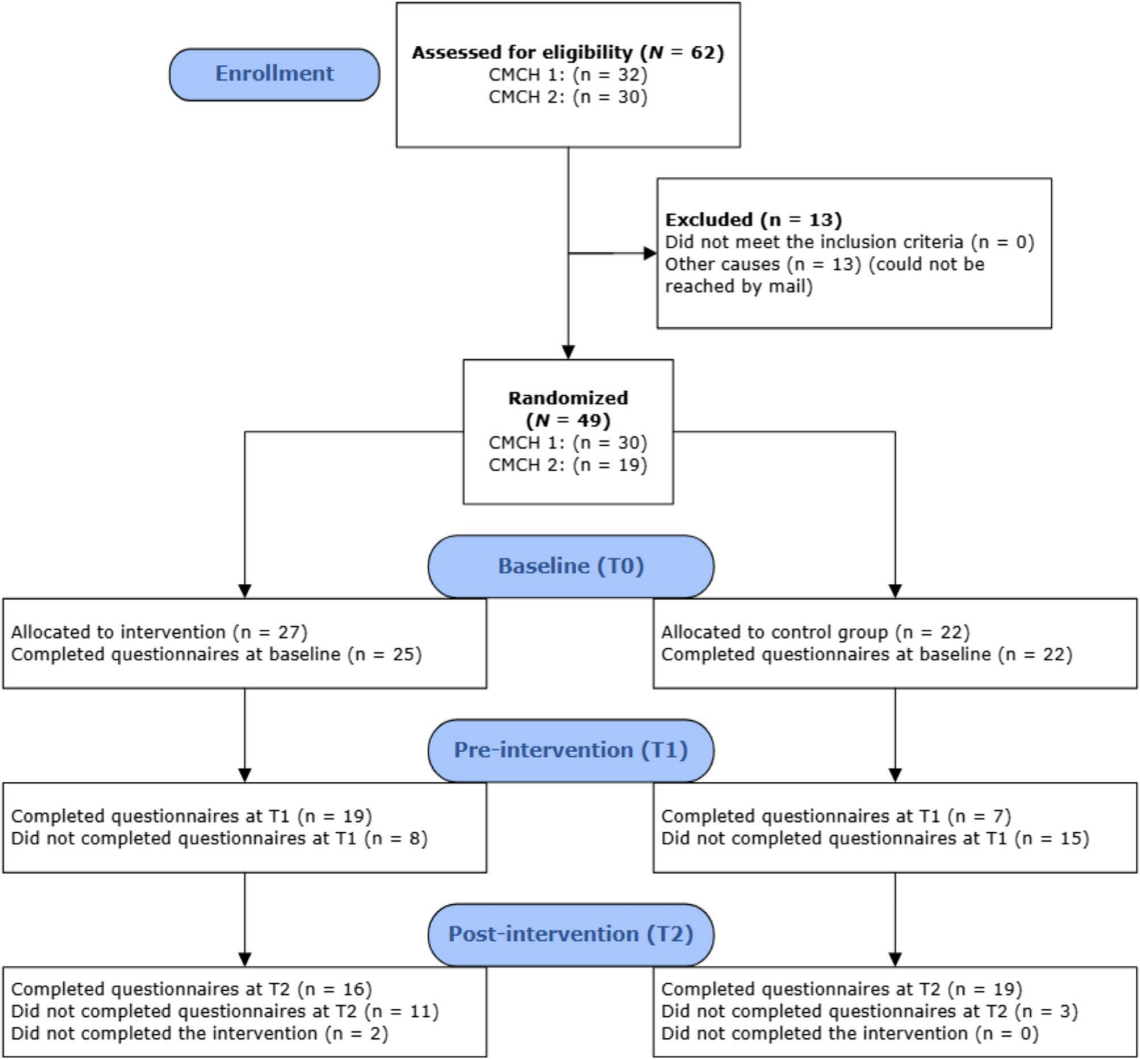
CONSORT flow diagram. Note: CMCH = Community mental health center, T0 = Time 0, baseline; T1 = Time 1, pre-intervention; T2 = Time 2, post-intervention, N = number of participants

Across the two study sites, 62 potential outpatients were invited to participate. Out of 62 patients eligible for inclusion, 49 patients agreed to participate in the study. The remaining 13 patients never responded to the study invitation sent by mail. Recruitment and randomization are presented in the consort diagram, Fig. 1.

Table 2 describes the sociodemographic characteristics. At baseline, independent samples *t*-tests and chi-squared test indicated no significant differences between the PG and CG.

**Table 2:** Baseline characteristics

Characteristics	PG, *n* = 27	CG, *n* = 22
Age:		
M (SD)	31.65 (8.648)	31.43 (8.072)
Range	20–60	20–49
Missing values	1 (3.7%)	1 (4.55%)
Sex:		
Male	5 (18.52%)	4 (18.18%)
Female	20 (74.07%)	18 (81.82%)
Missing values	2 (7.41%)	0
Relationships:		
Single	12 (44.44%)	12 (54.55%)
Married	4 (14.81%)	4 (18.81%)
Live with someone	7 (25.93%)	5 (22.73%)
Divorced	2 (7.41%)	1 (4.55%)
Missing values	2 (7.41%)	0
Educational level		
Primary/secondary school	7 (25.93%)	3 (13.64%)
Post-secondary school	12 (44.44%)	14 (63.64%)
High school/university	6 (22.22%)	5 (22.73%)
Missing values	2 (7.41%)	0
Employed/unemployed:		
Student	9 (33.33%)	3 (13.64%)
Employed	7 (25.93%)	8 (36.36%)
Employed partly (50%)	5 (18.51%)	3 (13.64%)
Disabled	2 (7.41%)	1 (4.55%)
Unemployed	1 (3.7%)	5 (22.73%)
Other	0	2 (9.09%)
Missing values	3 (11.11%)	0
Housing:		
Live alone	5 (18.51%)	5 (22.73%)
Live with parents	4 (14.81%)	2 (9.09%)
Live with only children	5 (18.51%)	3 (13.64%)
Live with spouse/cohabiting (and/or children)	10 (37.04%)	10 (45.45%)
Other	1(3.7%)	2 (9.09%)
Missing values	2 (7.4%)	0

*M* Mean, *n* Number of participants, *SD* Standard deviation, *PG* Psychoeducation group, *CG* Control group

### Quality of life

Between-group comparisons using linear mixed models showed a significant improvement in QoL. Participants in the PG had a significantly higher improvement in QoL as compared to the CG receiving TAU. The model-based difference between-groups at T2 was 6.90 (*p* = 0.04; 95% CI [0.20–13.60]). The effect size was moderate (Cohen’s *d* = 0.49), suggesting a meaningful improvement in perceived QoL among participants receiving psychoeducation, see Table 3.

**Table 3:** Linear mixed-effects model results and effect sizes for primary and secondary outcomes

Outcomes	Model-based Mean at T0	Model-based Mean at T1	Model-based Mean at T2		Model-based between-group differences at T2			Cohen’s d
			PG	CG	Diff	*p*	95% CI	
AAQoL	49.338	51.567	53.175	46.276	6.899	**0.044**	0.199–13.598	0.492
ASRS-6	16.277	15.679	15.710	16.143	− 0.433	0.638	−2.260–1.393	0.203
SIATS	9.660	9.458	11.617	9.478	2.139	**0.021**	0.328–3.951	0.782

Bolded *p*-values highlight statistically significant between-group differences

*T0* Time 0, baseline, *T1* Time 1, pre-intervention, *T2* Time 2, post-intervention, *PG* Psychoeducation group, *CG* Control group, *AAQoL* Adult ADHD Quality of Life Scale, higher scores indicate better QoL, *ASRS-6* Adult ADHD Self-Reported Scale, 6 Items, higher scores indicate more symptoms, *SIATS* Satisfaction with Information on ADHD and Treatment Scale, higher scores indicate greater satisfaction with information received, *95% CI* 95% confidence interval of the group difference at post-intervention, Cohen’s *d* indicates effect size: 0.2 = small, 0.5 = moderate, 0.8 = large

### ADHD symptoms and patient satisfaction

For ADHD symptom severity (ASRS-6), there was no statistically significant between-group difference (mean difference = −0.43; *p* = 0.64; 95% CI [−2.30–1.40]), and the effect size was small (Cohen’s *d* = 0.20).

Outpatients in the PG reported significantly higher satisfaction with the information on ADHD and its treatment, as measured by the SIATS (mean difference = 2.14; *p* = 0.02; 95% CI [0.33–3.60]). The effect size was large (Cohen’s *d* = 0.78), indicating that the psychoeducation program meaningfully improved participants’ satisfaction with the information they received compared to the CG (Table 3).

## Discussion

We conducted a pilot RCT of a novel psychoeducational program for adults with ADHD. This new psychoeducational group intervention was developed in collaboration between user representatives and healthcare professionals. The intervention was co-delivered in equal partnership by service users and clinicians. Between-group comparison, using linear mixed models, indicated a statistically significant improvement in QoL and satisfaction with information in the intervention group compared with the CG. The IG showed a moderate effect on QoL and a large effect on satisfaction with information relative to the CG. In contrast, the effect on ADHD symptom severity was non-significant, and the effect size was small.

The observed improvement in QoL in our study aligns with prior research on psychoeducation in adults with ADHD [7, 49, 51]. Vidal et al. [49] found that both group-based interventions, psychoeducation and cognitive-behavioral therapy, improved QoL, as measured by the Quality of Life Enjoyment and Satisfaction Questionnaire. Hoxhaj et al. [51] similarly reported significant QoL improvements among patients receiving either psychoeducation or mindfulness training (measured by the SF-36). Hirvikoski et al. [7] conducted an RCT comparing psychoeducational groups with TAU and observed significant improvements in QoL, as measured by the Satisfaction With Life Scale. By contrast, the open feasibility trial by Hirvikoski et al. [71] was the only study not to report QoL improvements, despite using the same QoL measure as in the present study. Although most studies suggest improvements in QoL, the findings are mixed, and the heterogeneity in QoL measures highlights the need to adopt a standardized QoL outcome measure.

In contrast to the observed improvement in QoL, no significant changes in ADHD symptoms were found between the PG and the CG, as measured by the ASRS-6. This finding aligns with previous studies [49, 51, 52] that, although reporting improvements in ADHD-related symptoms, did not demonstrate significant between-group differences. However, Selaskowski et al. [53] reported significant improvements in core symptoms in the intervention group. In that study, a traditional psychoeducation group was compared with a psychoeducation group supplemented by smartphone-assisted psychoeducation. This finding is noteworthy, as the use of a psychoeducational application can provide more accessible information over an extended period. In addition to traditional group sessions, smartphone applications may therefore be a valuable tool to support patients. Patient-centered education is fundamental to ensuring optimal health outcomes [72, 73], and the integration of digital tools presents an interesting angle for future studies on the delivery of education to patients. In the present pilot study, however, the lack of a significant improvement in ADHD symptoms may be due to the small sample size, which could have limited statistical power to detect between-group differences [74], or the limited responsiveness of the ASRS-6 used to measure ADHD symptoms. To reduce participant burden, particularly in a population with attention challenges, we used the ASRS-6, which has demonstrated good psychometric properties [62]. However, this choice may limit comparability with studies employing the full 18-item ASRS. Furthermore, while widely used, there is a lack of validation studies investigating this tool’s sensitivity in the Norwegian population. As such, the ASRS-6 may not be sensitive enough to detect subtle symptom changes over a brief period [75]. Future research should consider using measurement tools validated for the specific population to ensure the scale’s sensitivity [76].

Regarding satisfaction with the information, outpatients in the PG reported a significant increase in satisfaction with the information about ADHD and its treatment by the end of the intervention, possibly stemming from the increased understanding fostered by structured psychoeducation. Providing patients with structured information about ADHD, including its symptoms, causes, and treatment options, could empower patients to manage their condition while reducing uncertainty and anxiety [78]. Further, in our study, satisfaction with the information improved substantially in the PG compared with the CG, with a large effect size. This finding is consistent with Skliarova et al. [48]. Taken together with evidence from reviews [40, 47], feasibility studies [48, 71], and RCT-studies [54], these findings reinforce the value of psychoeducational group interventions in enhancing perceived knowledge and satisfaction.

The observed improvements in satisfaction with information and quality of life, on the one hand, and the lack of symptom reduction on the other, raise questions about the impact of psychoeducation and its use in clinical settings. Nevertheless, incorporating psychoeducation into the treatment of ADHD among adults aligns with current clinical guidelines [15, 77]. Furthermore, previous studies have shown that patients express a need for more detailed information about their diagnosis [34, 78]. Our baseline data and previous pilot studies [54] reflect similar concerns, highlighting the importance of structured psychoeducation. These findings suggest that standardizing the delivery of information could ensure that all outpatients receive consistent, clinically meaningful information. In addition, group-based psychoeducation offers the benefits of peer support [79], which can enhance the learning experience and improve overall satisfaction.

In our study, patients interacted with both healthcare workers and user representatives from an ADHD organization who provided practical advice and tips. This interactive group environment allowed the patients to relate to one another, making the information more meaningful and contributing to higher satisfaction levels [80]. Although this showed promising results, there are few studies examining these issues [40, 48], and larger studies are needed to draw conclusions on the specific impact of psychoeducational group programs.

### Strengths and limitations

There are several limitations to the current pilot RCT study. First, neither the outpatients nor the individuals administering the psychoeducational group intervention were blinded to the group allocations. To reduce potential biases, masked analyses were conducted, with the author responsible for analyzing the data (ÅH) blinded to the group allocations. This method was used to minimize any biases that could arise from knowing which participants were in the CG or PG. Second, as this was a pilot RCT study primarily designed to assess feasibility and generate preliminary data on QoL, the sample size was necessarily limited. Although this limitation restricts the generalizability of our findings, the study nonetheless provides an important foundation for the development of user-informed, patient-centered psychoeducational interventions for adults with ADHD. Third, the study was not powered to test hypotheses definitively. Therefore, all between-group comparisons should be interpreted as exploratory and intended to inform future research.

Fourth, the design is limited by using only three measurement points. Furthermore, although both T0 and T1 were collected before the intervention began, participants were treated as one single cohort during this period since neither group received any active treatment beyond usual care. Including both time points helped us assess whether outcomes were stable before the intervention and allowed us to focus our main comparison on the change from T1 to T2. This approach aimed to isolate the potential effect of the psychoeducation program. At the same time, we recognize that changes between T0 and T1, such as natural symptom variation or participant expectations, might have influenced results. This limitation highlights the need for careful planning of assessment timing in future studies. Designs using a single pre-treatment baseline or a more extended pre-intervention phase could help reduce this kind of variability. It’s also worth noting that the current study was limited to a pre-post design and did not include longer-term follow-up.

Fifth, in the present study, the overall dropout rate was low, with only two patients dropping out throughout the study. On the other hand, participants failed to complete the questionnaires, particularly in the CG. We interpret this missingness as likely due to random or logistical factors, rather than systematic bias related to group allocation or outcomes. However, to address potential bias, we conducted all analyses according to ITT principle, which preserves the advantages of randomization by including all participants in the groups to which they were originally assigned, regardless of missing data. Furthermore, linear mixed models were employed to accommodate missing-at-random data without imputation, preserving statistical power and reducing potential bias [81]. Finally, though the scale measuring the QoL was validated in a Norwegian setting [11], the scale measuring ADHD-related symptoms has not been validated in a Norwegian population. Additionally, it is a limitation that the SIATS has also not been validated. However, there is a lack of standardized outcome measures to assess satisfaction with information.

Despite the limitations mentioned above, this study also has several strengths. We believe that a key strength of the study is the involvement and collaboration of user representatives in the planning and execution of the intervention. This ensures that the intervention is relevant and tailored to patients diagnosed with ADHD in adulthood. Our results further add to a growing but still limited body of evidence that patient-centered psychoeducation can improve informational and experiential aspects of care, even when core symptom scores show little immediate change. Other strengths include the RCT design, the limited exclusion criteria, the real-life setting, and the similar baseline characteristics between the PG and CG, which provide a good starting point for comparison and drawing conclusions.

## Conclusion

This pilot RCT provides support for the use of a co-delivered psychoeducational group intervention in improving quality of life and satisfaction with information among adults newly diagnosed with ADHD. However, no significant improvement in ADHD-related symptoms was observed. Future research should build on these findings by employing larger samples, longer follow-up periods and standardized outcome measures.

## Supplementary Information

Supplementary Material 1.

## Data Availability

The dataset that supports the findings of the current study is not publicly available due to ethical and legal reasons. However, the data supporting the findings of this pilot study are available from the corresponding authors upon reasonable request and with permission from the ethical committee.

## Abbreviations

AAQoL: Adult ADHD Quality of Life Scale
ADHD: Attention-deficit/hyperactivity disorder
ASRS-6: Adult ADHD Self-Reported Scale, 6 Items
CG: Control group
CI: Confidence interval
CMCH: Community mental health center
CONSORT: Consolidated Standards of Reporting Trials
*d*: Effect size, Cohen’s d
ITT: Intention-to-treat
*M*: Mean
*MD*: Mean difference
PG: Psychoeducation group
RCT: Randomized controlled trial
SD: Standard deviation
SIATS: Satisfaction with Information on ADHD and Treatment Scale
T0: Time 0, baseline
T1: Time 1, pre-intervention
T2: Time 2, post-intervention
TAU: Treatment as usual
QoL: Quality of life
